# Spatial Heterogeneity of Habitat Selection of Large Carnivores and Their Ungulate Prey in Proximity to Roads

**DOI:** 10.1002/ece3.70971

**Published:** 2025-02-10

**Authors:** Xuankai Liang, Zexu Long, Shiyu Chen, Jinzhe Qi, Buyi Sun, Nathan James Roberts, Heng Bao, Guangshun Jiang

**Affiliations:** ^1^ Feline Research Center of National Forestry and Grassland Administration, College of Wildlife and Protected Area Northeast Forestry University Harbin China; ^2^ Northeast Asia Biodiversity Research Center Northeast Forestry University Harbin China; ^3^ Bioscience College Guizhou Education University Guiyang China; ^4^ Central European University Vienna Austria

**Keywords:** Amur leopard, Amur tiger, geographically weighted regression, habitat selection, predator–prey, spatial heterogeneity

## Abstract

Geographic heterogeneity, encompassing both species‐environment interactions and interspecific relationships, significantly influences the ecological attributes of wildlife habitat selection and population distribution. However, the impact of geographic heterogeneity on the distribution of target species within predator–prey systems, particularly in human‐dominated landscapes, remains unclear. By conducting line transect surveys, utilizing a monitoring network, and applying logistic geographically weighted regression (GWR) in conjunction with generalized linear models (GLM), we examined the spatial heterogeneity of habitat selection by the Amur tiger, Amur leopard, and their main ungulate prey, wild boar and roe deer, in Northeast China. Our results suggest that the factors affecting the spatial distribution of predators are more complex than those for prey. More significantly, the selection coefficients of roe deer and wild boar for certain habitat factors serve as crucial explanatory variables in the Amur tiger and leopard models. Our findings emphasize the importance of spatial non‐stationarity in predator–prey habitat selections, and the heterogeneous selection by prey may drive dispersals of large felids across complex road landscapes. This study offers new insights into how to help apex predators cross road barriers by effectively managing prey habitat selection in a landscape dominated by roads, providing valuable guidance for future habitat conservation policies.

## Graphical abstract

Based on line transect survey and a monitoring network, this study examined the spatial heterogeneity of habitat selection of Amur tiger and leopard, as large carnivores, and their main ungulate prey, wild boar and roe deer, in Northeast China, by applying logistic geographically weighted regression (GWR) in conjunction with generalized linear models (GLM).

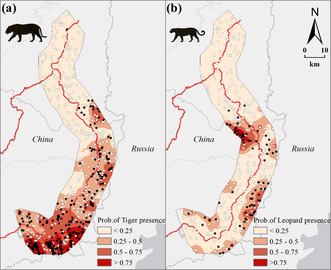

## 1 Introduction

Understanding the ecological determinants of species distribution and abundance has always been a key area of wildlife ecology, conservation, and management (Andrewartha and Birch 1954; Pease et al. 2022). Species distributions are driven by various biotic and abiotic factors and their interactions at varying temporal and spatial scales (Liu et al. 2019). The properties of habitat variables of concern experience substantial transformations as the impacts of various factors on ecological processes vary across different temporal and spatial scales (Phillips et al. 2006). These varying response properties may differ across space and time, commonly referred to as non‐stationarity (Rollinson et al. 2021). Further, human disturbance and the resulting changes in natural landscape patterns, climates, and other factors make it full of challenges to explore such associations, especially when considering the geographical heterogeneity of both species‐environment and inter‐specific relationships (Foley et al. 2005; Guisan and Thuiller 2005).

Large carnivores play significant roles in ecosystem development and regulation (Ripple et al. 2014). Habitat selection, interspecific relationship with prey, and response to anthropogenic disturbances have consistently been the focus of conservation and management practice for large carnivores (Bhattarai and Kindlmann 2012; Kafley et al. 2019; Wang et al. 2016). As predators, their distribution and population structure are largely determined by the distribution and abundance of their prey (Karanth et al. 2004; Mitchell and Hebblewhite 2012). Meanwhile, as the most common and widespread anthropogenic disturbance, roads exhibit the most intricate ramifications on large carnivores, including substantial direct impact on individual mortality through increasing roadkill as well as serious indirect influences, such as habitat fragmentation, potentially facilitating enhanced hunting access that can lead to diminished prey availability (Kerley et al. 2002; Torres et al. 2016).

Species Distribution Models (SDMs), also known as ecological niche models or habitat suitability models, are an analysis technique commonly applied in biogeography and ecology (Elith and Leathwick 2009). SDMs are constructed based on ecological niche theory and usually combine observation of species occurrence or abundance with environmental predictors to estimate the probability of species occurrence or species range (Elith and Leathwick 2009). Additionally, SDMs are commonly used to understand relationships between species occurrence and habitat factors (Bakka et al. 2016; Maryam et al. 2018). One prerequisite when using SDMs is the assumption that associations between environmental predictors and species occurrence are constant across space and time in the study area, called stationarity (Miller 2012). Factors like sampling variation, an intrinsically different process in different parts of the study area, and a model with a missing variable may result in non‐stationarity (O'Sullivan 2003).

Many models frequently used, such as generalized linear models (GLMs) (Nelder and Wedderburn 1972), assume that the environmental factors affect species similarly across the whole study area, and estimate a constant coefficient using maximum likelihood. However, this assumption may be inconsistent with situations in regions with high heterogeneity. Alternatively, geographically weighted regression (GWR) (Brunsdon et al. 1996) estimates one unique coefficient for each sampled location to meet a spatial non‐stationarity assumption (Brunsdon et al. 1996; Byrne et al. 2009), addressing the flaw in global regression models (Byrne et al. 2009) and providing a different approach to better understand species‐environment associations and their geographical heterogeneity (Lauri et al. 2014; Liu et al. 2016; Windle et al. 2010). For instance, GWR has been successfully applied to investigate spatial non‐stationary of environmental effects on giant pandas ( *Ailuropoda melanoleuca* ) distribution (Ye et al. 2020), and to investigate effects of local spatial heterogeneity on multivariate relationships of white‐tailed deer ( *Odocoileus virginianus* ) distribution (Haijin et al. 2005). The limitation of GWR is that it cannot be used for estimating, extrapolating or predicting to a new place or time since it is against the non‐stationarity assumption (Hothorn et al. 2011; Li et al. 2018; Osborne et al. 2010). However, this restriction could be realized by integrating GLM and logistic GWR (Mirbagheri and Alimohammadi 2017). This integration not only allows additional and prediction processes but also provides better explanations for the non‐stationarity assumption because multiple models lead to the estimation of multiple unique parameters (Behan et al. 2021).

Amur tiger ( *Panthera tigris altaica* ) and leopard ( *Panthera pardus orientalis* ) are both endangered large carnivores distributed in Northeast China and the Russian Far East. Ungulates, like wild boar ( *Sus scrofa* ), sika deer ( *Cervus nippon* ), and roe deer ( *Capreolus pygargus* ) are their main prey (Gu et al. 2018; Yang et al. 2018). Many studies have tried to explore the effects of biotic and abiotic factors on the spatial distribution of Amur tiger and leopard (Carroll and Miquelle 2006; Jiang et al. 2015; Long et al. 2021), and it has been identified that prey and anthropogenic disturbances have substantial impacts on their distribution (Wang et al. 2016; Woodroffe 2000). However, all of these studies used “spatial stationary models”, such as GLMs (Jiang et al. 2015), GAMs (Carroll and Miquelle 2006), and Maxent (Long et al. 2021), which may have overlooked the reality of geographical heterogeneity (Yackulic 2016).

In this study, we employed the GWR in conjunction with GLM to examine the spatial heterogeneity of habitat selection by the Amur tiger, Amur leopard, and their ungulate prey in road‐dominated landscapes in Northeast China. Furthermore, we seek to investigate whether the habitat selection of ungulate prey influences the preference of Amur tigers and Amur leopards in these habitats close to roads. Specifically, we hypothesized that (1) in forest landscapes with high local heterogeneity, non‐stationarity of habitat selection is common to most species; (2) geographic heterogeneity of ungulate habitat selection affects the responses of tigers and leopards to roads, these new insights could aid the mitigation of road impacts on large carnivores.

## 2 Material and Methods

### 2.1 Study Area

**FIGURE 1:**
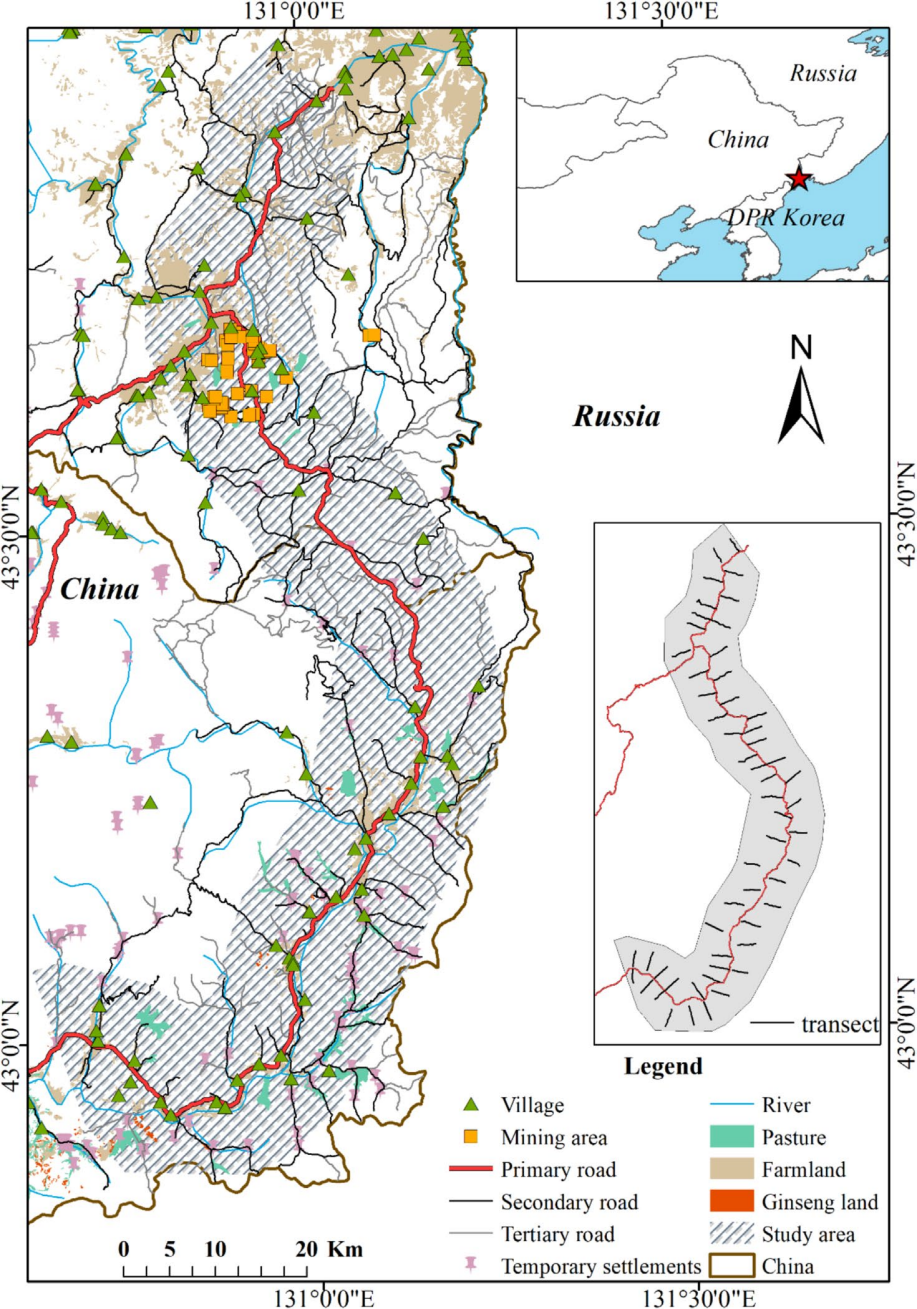
Geographical maps, human activities, and the placement of sampling transects within the study.

The study area is located in the Northeast Tiger and Leopard National Park (NTLNP) of China. We mainly focused on national highway G331 in the NTLNP, which shows an obvious barrier effect on the migration and dispersal of the Amur tiger and leopard. We applied an 8‐km width buffer to the G331 and clipped it within the boundary of NTLNP using ArcMap v10.8 (42°52′6″–43°59′5″ N, 130°37′5″–131°15′41″ E; Figure 1). The length of the G331 inside the NTLNP is approximately 186.93 km, connecting several villages and towns, including larger settlements like Laoheishan and Chunhua. There is a notable difference in anthropogenic disturbance between the southern and northern regions of the study area due to varying local living and production style; mining areas are predominantly concentrated in the northern region, while pasture and ginseng land are primary land use in the southern region (Figure 1).

### 2.2 Species Distribution Data

The occurrences of Amur tigers (*n* = 352) and leopards (*n* = 273) in the study area were derived from a continuous comprehensive monitoring network from 2012 to 2018. We collected the occurrence data of Amur tigers and leopards by integrating camera traps, livestock predation records, snow track surveys, kill site investigations, footprint identification, and feces or hair samples collection (Qi et al. 2021). These occurrence data were collected opportunistically rather than systematically, so they were regarded as presence‐only data. In the winter of 2017–2018, we deployed 55 snow transects, each with a minimum length of 5 km, on each side of the G331; the total length of transects was 287 km. The survey involved a total of 31 participants, with 2 individuals, one professional person with the skill to identify animal footprints on the snow and a local guide, assigned to each transect; notably, each transect was surveyed only once. These transects were evenly distributed, with an average distance of 3 km between any two adjacent transects (Figure 1). The survey involved recording detailed information on ungulate occurrences, including footprints, feces, urine marks, and resting sites (< 24 h). We collected 39 records of sika deer, 4 records of red deer, 414 records of roe deer, and 89 records of wild boar. Sika deer and red deer were removed from analysis due to too few occurrence points for robust analysis.

### 2.3 Environmental Variables

We selected a set of 21 geographic information system (GIS) based environmental factors as initial potential predictors (see Table A1 for details), which have been commonly used in assessing the habitats of Amur tigers, Amur leopards, and their ungulate prey (Hebblewhite et al. 2014; Jiang et al. 2014; Qi et al. 2015; Xiaofeng et al. 2011). All these potential predictors were classified into five types: topographic factors, forest cover, human disturbance, snow depth, and food abundance. Continuous factors, for example, elevation, slope, snow depth, and number of branches of the current year's shrubs, were calculated as the average within a focal cell. Categorical factors, such as forest type, land use, and human disturbance, were quantified by calculating the distance from occurrence points to each site of these categories. All variables were resampled to a grid size of 200 m using bilinear interpolation (Mastyo 2013) within ArcGIS (ESRI, v. 10.8).

### 2.4 Model Development

**FIGURE 2:**
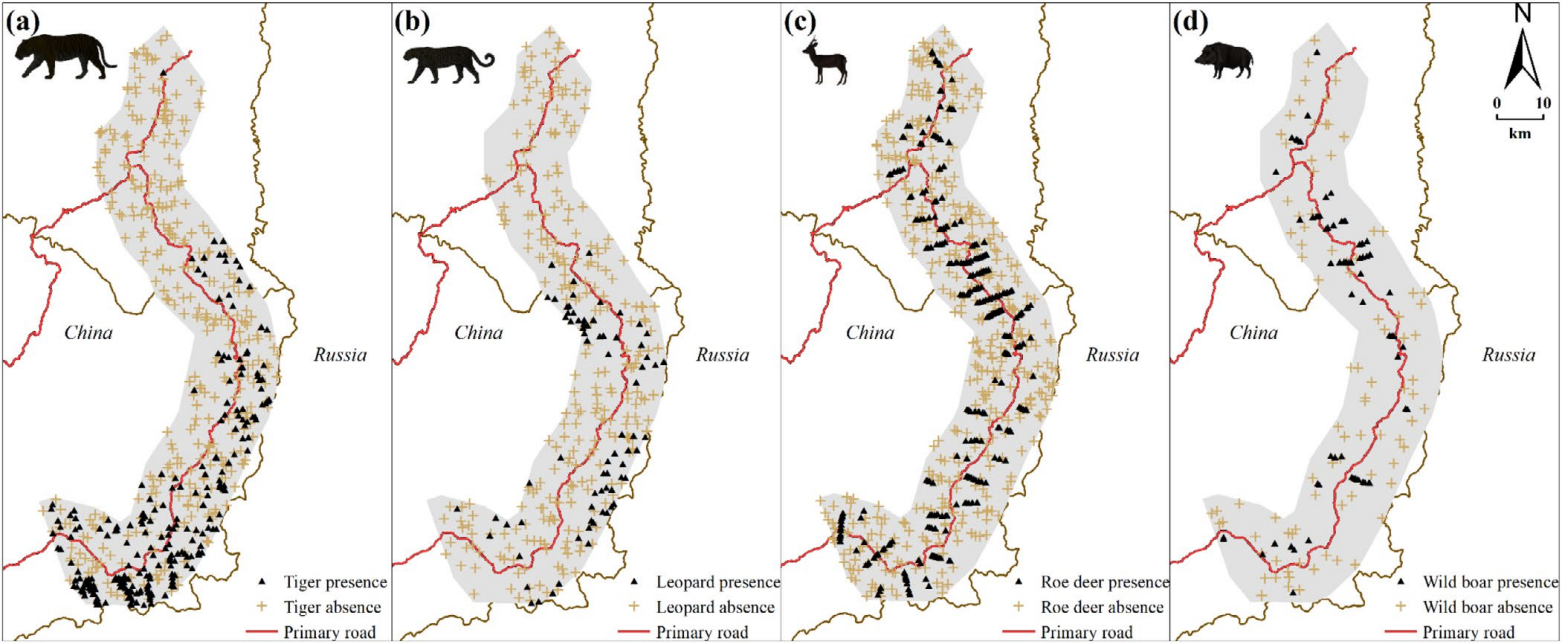
Presence samples of Amur tigers (a) and Amur leopards (b, filled triangles), along with pseudo‐absence samples (plus signs), in the study area were derived from a continuous comprehensive monitoring network 2012–2018, consisting of camera traps, livestock predation records, snow track surveys, kill site investigations, footprint identification, and feces or hair samples collection. Furthermore, roe deer (c) and wild boar (d) samples were obtained through snow track line transect surveys conducted in the winter of 2017–2018.

We randomly generated an equal number of pseudo‐absences points outside a 200 m buffer zone of the occurrence data points (Barbet‐Massin et al. 2012). Predator occurrence data were obtained opportunistically and suffered from sampling bias. Thus, one way to mitigate this issue is to filter occurrence data spatially (Boria et al. 2014). We applied a spatial filter with a distance threshold of 200 m to both presence and pseudo‐absence locations using a Python‐based GIS toolkit “SDMtoolbox” (Brown 2014). Our final dataset consisted of presence records (and pseudo‐absence in parentheses) as: Amur tiger 247 (347); Amur leopard 73 (267); roedeer 265 (414), and; wild boar 67 (89) (Figure 2). These presence and pseudo‐absence records were utilized as binary response variables in species distribution modeling.

To make coefficients of environmental factors comparable (Zuur et al. 2010), all environmental predictors were standardized by z‐transformation before modeling. To reduce multicollinearity among potential predictors, we statistically thinned the preliminary set of environmental variables by the “corSelect” function in the “fuzzySim” R package (Barbosa 2015). Variance inflation factor (VIF) indexes were estimated for multicollinearity diagnosis. If the correlation coefficient of a pair of variables was > 0.75 or the VIF values were > 10 (Zuur et al. 2010), the variables were tested in a bivariate model and the one with a better fit was retained.

Subsequently, GLM was employed to identify the optimal combination of models by selecting the full model which incorporated the aforementioned variables. This study employed methods from the “MuMIn” R package (Barton 2013) to perform model selection based on the full model, to determine the best set of potential predictors that influence the occurrences of each species (Anderson and Burnham 2004). The “dredge” function implemented a subset regression approach by generating all possible subsets of models and ranking them based on criteria such as AIC values, helping to ultimately select the optimal subset. Secondly, we applied logistic GWR to explore the spatial non‐stationarity of the relationship between environment factors and the spatial distribution of target species. Logistic GWR analysis was performed with GWR 4.0 software (available at https://gwr.maynoothuniversity.ie/gwr4‐software/). The basic GWR model, provided by Fotheringham et al. (2003), is explained as follows.

$$\left[\frac{y_{i}^{*}}{1-y_{i}^{*}}\right]=\beta_{0i}+\sum_{k}\beta_{\mathrm{ki}}x_{\mathrm{ki}}+\varepsilon_{i}$$

where $y_{i}^{*}$ is the probability that a species may occur at point i, $\beta_{0i}$ is the intercept specific to observation i, and $\beta_{\mathrm{ki}}$ is the coefficient for the $k^{\mathrm{th}}$ covariate at observation i, and $x_{\mathrm{ki}}$ and $\varepsilon_{i}$ are the $k^{\mathrm{th}}$ covariate and error at observation i, respectively.

When modeling the distribution of the Amur tiger and Amur leopard, we also applied the selection coefficients of each ungulate species in relation to significant various environmental factors as potential predictors. This approach allows us to assess how the habitat preferences of prey species influence the distribution and behavior of these apex predators.

### 2.5 | Bandwidth Selection

In a logistic GWR model, local variable coefficients for each observation are fitted via a distance‐decay kernel weighting scheme where neighbors closer to the modeled observation are weighted more heavily than those further away (Fotheringham et al. 2003). In practical applications, selecting the kernel bandwidth of the weighting function is critical for model performance. Two primary methods can be employed: “fixed” and “adaptive.” A fixed kernel bandwidth implies that the distance threshold used to compute the weights remains constant across the entire study area. This method is appropriate for scenarios where the spatial distribution is relatively uniform. However, for datasets characterized by non‐uniform spatial distributions, such as species distribution in nature reserves, a fixed kernel bandwidth can lead to overfitting in densely populated areas and underfitting in sparsely populated regions (Atkinson et al. 2003; Nakaya et al. 2005). In contrast, the adaptive kernel bandwidth provides enhanced flexibility (Brunsdon et al. 1996). It dynamically adjusts its size according to the data density around each focal point. In sparse data areas, the adaptive kernel uses a larger bandwidth to include enough neighboring points, reducing bias from small samples. In dense data areas, a smaller bandwidth enhances local fitting accuracy and captures subtle variations (Fotheringham et al. 2003).

Given the evident irregularity in the distribution of species presence‐absence points in this study, we selected the Gaussian spatially weighted function with an adaptive kernel as the foundation of the logistic GWR model. To ascertain the optimal bandwidth size, we employed the method of minimizing the corrected Akaike Information Criterion (AICc). This approach not only effectively addresses the complexity of data distribution but also aligns with the recommended practices outlined by Fotheringham et al. (2003), thus enhancing the accuracy and interpretability of the model.

### 2.6 | Model Fitting and Validation

Logistic GWR and GLM performance was examined based on AICc and adjusted *R* ^2^ (Miller 2012). General, when two models have a difference in AICc of greater than four, they are considered to have different goodness of fits (Saefuddin et al. 2012). We calculated the Area under the receiver operating characteristic curve (AUC) to test the ability of the model to discriminate species presences from pseudo‐absences (Zou et al. 2007), which plots sensitivity against (1‐specificity) over a number of classification thresholds (Gregory et al. 2007). AUC values exceeding 0.9 indicate excellent model performance, values between 0.7 and 0.9 indicate useful models, and other values are considered flawed or erroneous. Additionally, we calculated and compared the True Skill Statistic (TSS) of each model, which is not sensitive to species occurrence (Allouche et al. 2006): TSS value ranging from 0.6 to 0.8 were considered useful, and values higher than 0.8 were excellent.

We used the subsample method to assess model validation performance. The original species data (presence and pseudo‐absence) were randomly sampled 30% without replacement for model validation, the remaining was used for model calibration. This process was repeated 5 times, and AUC and TSS values were averaged. Statistical analyses of this research were conducted in the R environment (Team 2013) using the “pROC” package (Robin et al. 2011).

### 2.7 | Mapping Spatial Heterogeneity of Species‐Environment and Predator–Prey Relationships

We evaluated the geographic heterogeneity of selectivity of Amur tigers, leopards, and their ungulate prey for habitat variables in the study area. Subsequently, significant local coefficients derived from logistic GWR models for each species were transformed into continuous surfaces by using Inverse Distance Weighting (IDW) interpolation (Windle et al. 2010). All continuous surfaces of local parameters were plotted in ArcGIS (ESRI, v10.8).

## 3 Results

### 3.1 Ungulate Prey GLM and Logistic GWR Models

Our GLMs shows that human disturbance and forest cover are the main factors affecting the spatial distribution of roe deer and wild boar. Seven environment factors related to roads and vegetation formed the optimal GLM of roe deer: distance to primary road (P_Road), distance to secondary road (S_Road), distance to tertiary road (T_Road), current year's shrubs (Food), distance to broadleaved deciduous forest (BDF), distance to open mixed broadleaved and needle‐leaved forest (MBNF), and distance to mosaic forest or shrubland/grassland (MFSG). Significant predictors of wild boar distribution include distance to temporary settlements (Settlements), distance to pasture (Pasture), P_Road, T_Road, snow depth (Snow), distance to open needle‐leaved deciduous or evergreen forest (NDEF), and MFSG.

**FIGURE 3:**
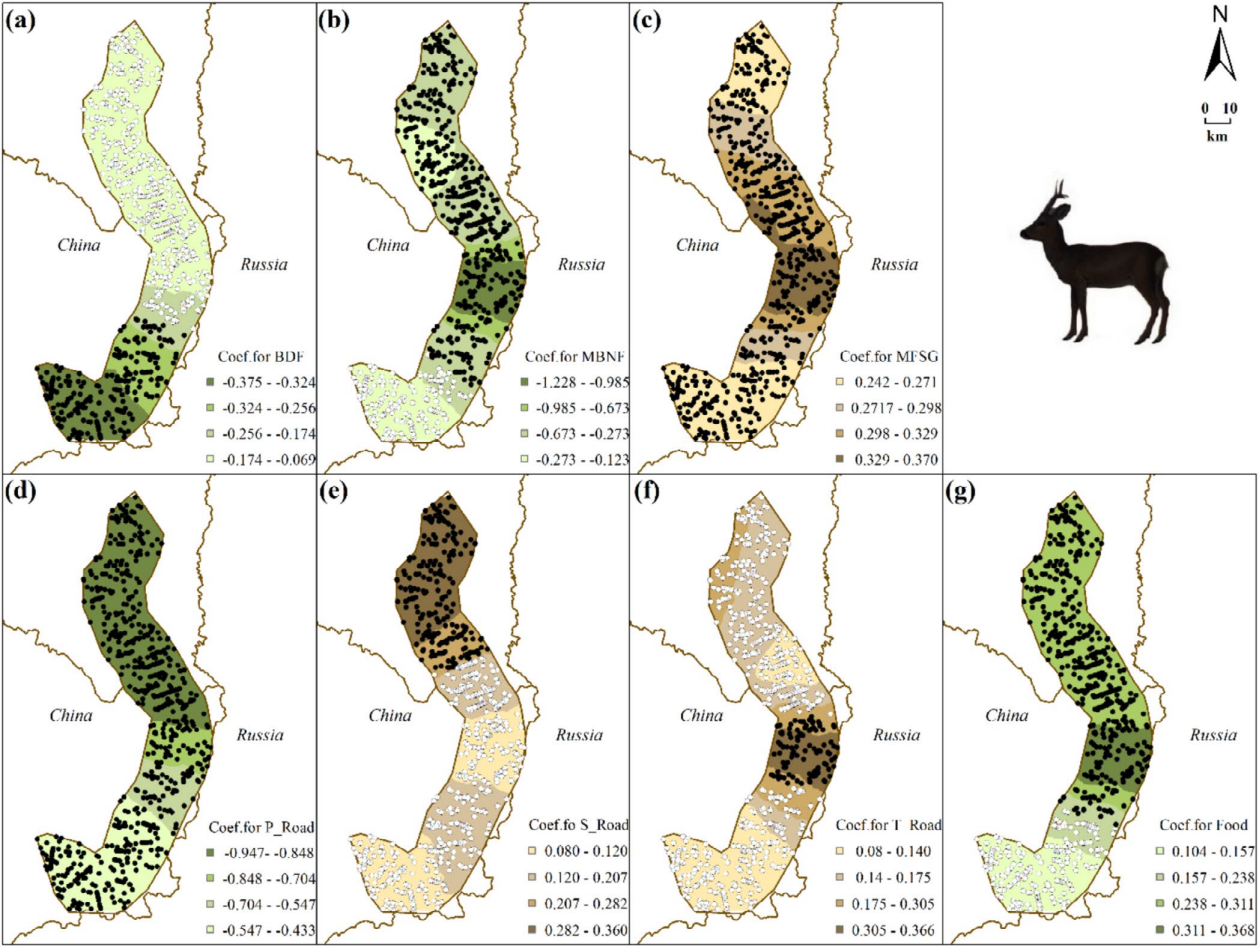
Interpolated continuous surfaces of local coefficient estimate of variables derived from roe deer logistic GWR: regarding the color selection of the base map: Green signifies preference, whereas brown denotes avoidance. Filled circles represent samples where a significant relationship between roe deer distribution and each variable was observed at *p* < 0.05 level, while unfilled circles indicate non‐significant samples. (a) BDF: Distance to closed broadleaved deciduous forest patch, (b) MBNF: distance to closed to open mixed broadleaved and needle‐leaved forest patch, (c) MFSG: Distance to mosaic forest or shrubland/grassland patch, (d) distance to primary road, (e) distance to secondary road, (f) distance to tertiary road, and (g) food abundance measured as the number of branches on current year's shrubs within a 200 m grid.

**FIGURE 4:**
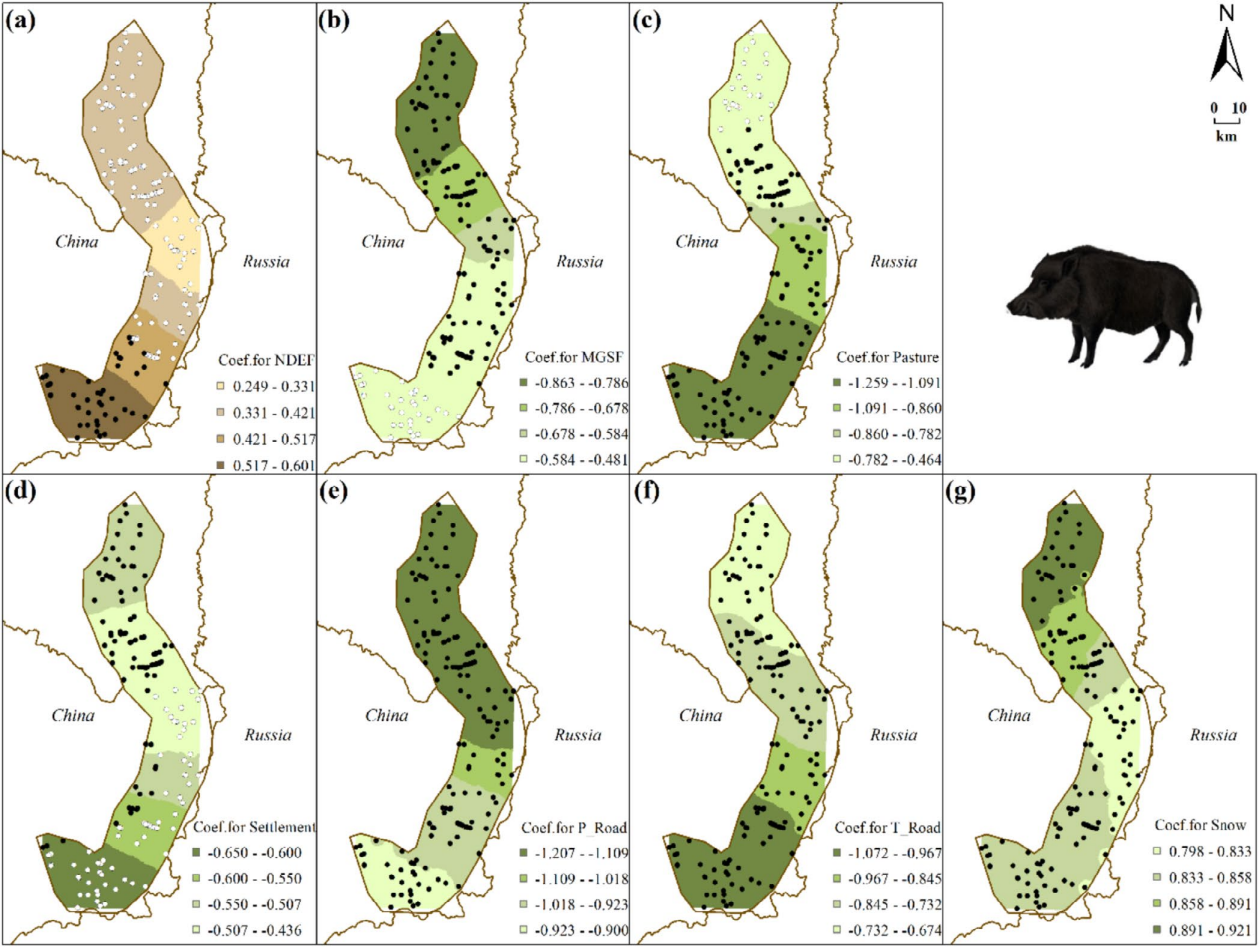
Interpolated continuous surfaces of local coefficient estimate of variables derived from wild boar logistic GWR: Regarding the color selection of the base map: Green signifies preference, whereas brown denotes avoidance. Filled circles indicate significant relationships between wild boar distribution and the respective variable at a significance level of *p* < 0.05, while unfilled circles represent non‐significant samples. (a) NDEF: distance to patches of open needle‐leaved deciduous or evergreen forest, (b) MGSF: distance to patches of mosaic grassland/forest or shrubland, (c) distance to pasture areas, (d) distance to temporary settlements, (e) distance to primary roads, (f) distance to tertiary roads, and (g) snow depth—representing average snow depth within a 200 m grid.

Logistic GWR models (roe deer model: TSS = 0.403, AUC = 0.742; wild boar model: TSS = 0.645, AUC = 0.839) show the spatial heterogeneity of species‐environment relationships (Table 1; Figures 3 and 4, see Table A2 for details). MFSG and P_Road have significant impacts on roe deer distribution across the study area (Figure 3c,d). S_Road only demonstrates a significantly positive correlation with roe deer distribution in the northern part (Figure 3e), whereas T_Road only exhibits a significantly positive correlation in the central part (Figure 3f). For wild boar, factors such as P_Road, T_Road, and Snow significantly impact wild boar distribution across the study area (Figure 4e–g). Specifically, both P_Road and T_Road show significant negative correlations with wild boar distribution. Additionally, snow depth positively correlates with wild boar distribution throughout the study area (Figure 4g).

**TABLE 1:** Summary of generalized linear models (GLM) and logistic geographically weighted regression (logistic GWR) model performances of predator and prey distribution.

Species	Model	GLM				Logistic GWR			
		AICc	Pseudo R^2^	TSS	AUC	AICc	Pseudo *R* ^2^	TSS	AUC
Roe deer	P _ Road + S _ Road + T _ Road + Food + BDF + MBNF + MFSG	811	0.121	0.358	0.722	799	0.154	0.403	0.742
Wild boar	Settlements + Pasture + P _ Road + T _ Road + Snow + NDEF + MGFS	173	0.263	0.574	0.828	175	0.292	0.645	0.839
Tiger	Slope + Farmland + Mining + P _ Road + NDEF + R_MBNF + W _ Snow	583	0.298	0.522	0.838	572	0.353	0.564	0.864
Leopard	Aspect + Settlement + Farmland + P _ Road + T _ Road+ R _ S _ Road	245	0.346	0.616	0.879	246	0.357	0.627	0.884

*Note:* P _ Road indicates distance to primary road, S _ Road indicates distance to secondary road, T _ Road indicates distance to tertiary road, Food indicates number of branches of the current year's shrubs in a 200 m grid. Pasture indicates the distance to pasture, Settlements indicate the distance to temporary settlements, and Snow indicates average snow depth within a 200 m grid, Farmland indicates the distance to farmland, BDF indicates the distance to a closed broadleaved deciduous forest patch, MBNF indicates the distance to closed to open mixed broadleaved and needle‐leaved forest patch, MFSG indicates the distance to mosaic forest or shrubland/grassland patch, NDEF indicates the distance to open needle‐leaved deciduous or evergreen forest patch, MGFS indicates the distance to close to mosaic grassland/forest or shrubland patch, R_MBNF indicates the local coefficient of the MBNF variable in the logistic GWR model for roe dee distribution, W _ Snow indicates the local coefficient of the Snow variable in the logistic GWR model for wild boar distribution, R _ S _ Road indicates the local coefficient of the S _ Road variable in the logistic GWR model for roe dee distribution.

### 3.2 Predator GLM and Logistic GWR Models

**FIGURE 5:**
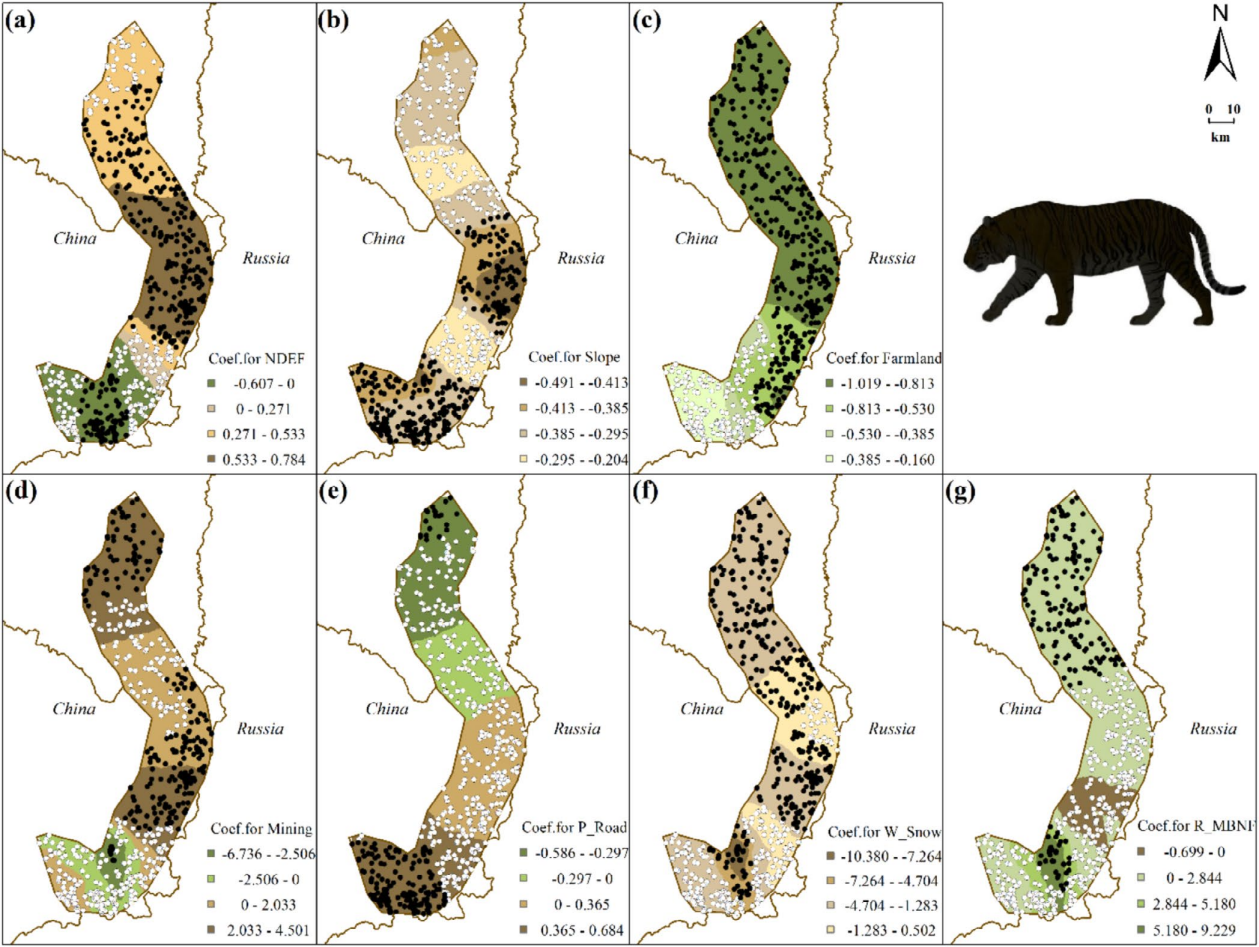
Interpolated continuous surfaces of the local coefficient estimate of variables derived from tiger logistic GWR: Regarding the color selection of the base map: Green signifies preference, whereas brown denotes avoidance. Filled circles represent statistically significant relationships between Amur tiger distribution and respective variables at *p* < 0.05 level; unfilled circles indicate non‐significant samples. (a) NDEF: distance to patches of open needle‐leaved deciduous or evergreen forest, (b) slope, (c) distance to farmland, (d) distance to mining areas, (e) distance to primary roads, (f) W_Snow: local coefficient of the Snow (snow depth—representing average snow depth within a 200 m grid) variable in the logistic GWR model for wild boar distribution, and (g) R_MBNF: Local coefficient of the MBNF (distance to closed to open mixed broadleaved and needle‐leaved forest patch) variable in the logistic GWR model for roe deer distribution.

**FIGURE 6:**
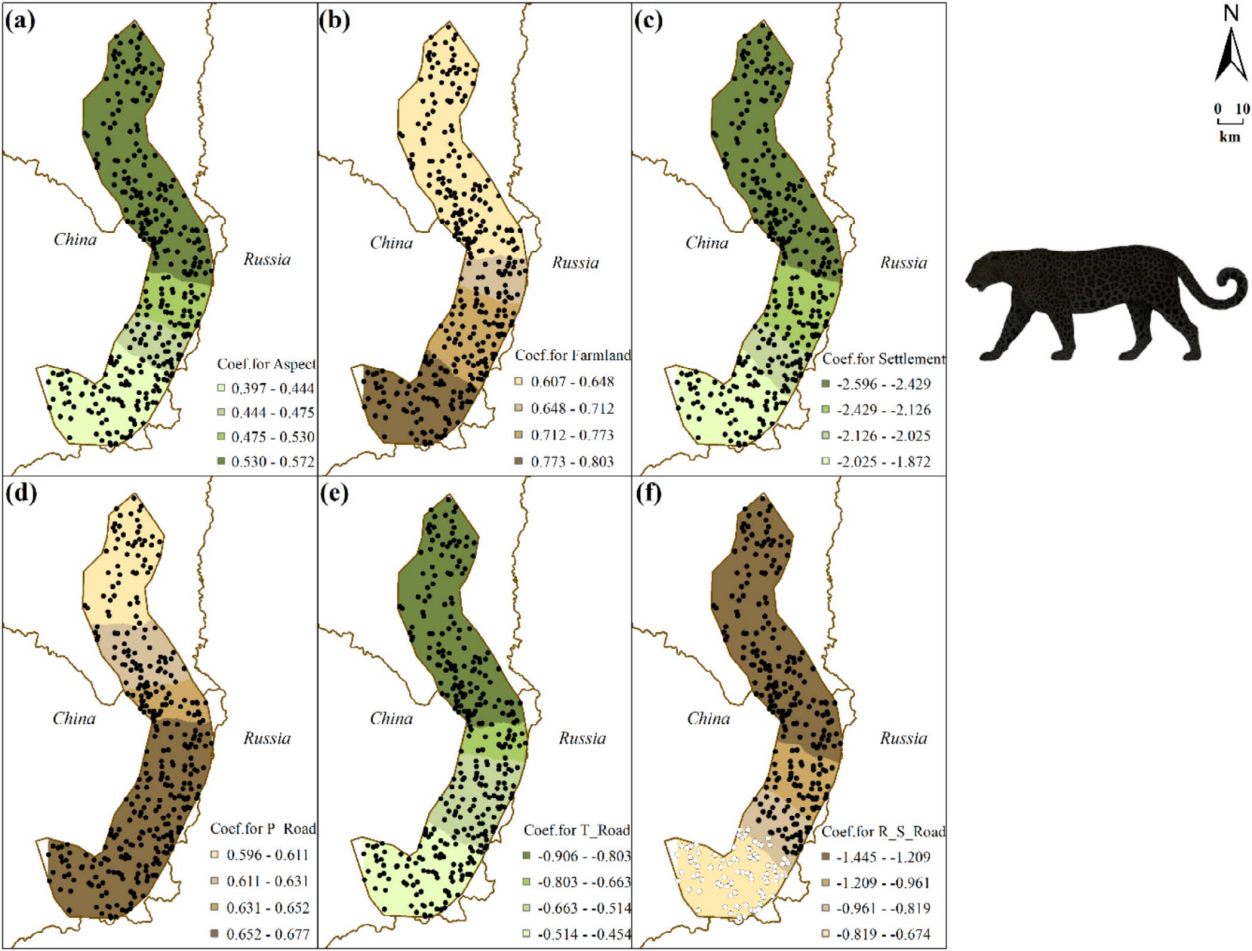
Interpolated continuous surfaces of the local coefficient estimate of variables derived from leopard logistic GWR: Regarding the color selection of the base map: Green signifies preference, whereas brown denotes avoidance. Filled circles represent statistically significant relationships between Amur leopard distribution and respective variables at *p* < 0.05 level; unfilled circles indicate non‐significant samples. (a) aspect, (b) distance to farmland, (c) distance to settlement, (d) distance to primary road, (e) distance to tertiary road, and (f) R_S_Road: Local coefficient of the S _ Road (distance to secondary road) variable in the logistic GWR model for roe dee distribution.

The composition of factors explaining the spatial distribution of Amur tiger and leopard is more complex than that of roe deer and wild boar (Table 1, see Table A2 for details). Topographic factors, forest cover, human disturbance, and prey all play a significant role in this process. More importantly, the selection coefficients of roe deer and wild boar, as the main prey, for some habitat factors are important explanatory variables in the models of Amur tiger and leopard (Figures 5 and 6, see Table A2 for details).

The tiger distribution logistic GWR model (TSS = 0.564, AUC = 0.864, Figure 5) exhibited a high degree of spatial heterogeneity across the study area, showing a significant negative correlation with local coefficient of the snow variable in the logistic GWR model for wild boar distribution(W_Snow), except for certain areas in the central and southern regions (Figure 5f). Conversely, there is a significant positive correlation between tiger distribution and local coefficient of the MBNF variable in the logistic GWR model for roe deer distribution(R_MBNF) in the northern and southern regions (Figure 5g). Variables such as NDEF, Mining, P_Road, and R_MBNF display exact opposite effects on the distribution of tigers in different areas (Figure 5). For instance, Mining is positively correlated with tigers in the central and northern areas but negatively correlated with tiger in certain smaller southern regions. Furthermore, in the southern region, P_Road demonstrates a significant positive correlation with tiger presence, while no significant correlation is observed in most of the northern region (Figure 5e).

Based on the leopard logistic GWR (TSS = 0.627, AUC = 0.884, Figure 6), aspect exhibits a significant positive correlation with leopard occurrences within the study area, gradually diminishing from north to south. Farmland and P_Road also demonstrate noteworthy positive associations with leopard presence, intensifying from north to south (Figure 6b). Conversely, Settlements and T_Road exhibit substantial negative correlations with leopard presence, attenuating from north to south. Notably, local coefficient of the S _ Road variable in the logistic GWR model for roe dee distribution (R_S_Road) shows a significant negative correlation with leopard occurrence across most of the study area, except for certain areas in the southern part (Figure 6f).

### 3.3 Probability of Tiger and Leopard Presence Predicted by Logistic GWR

**FIGURE 7:**
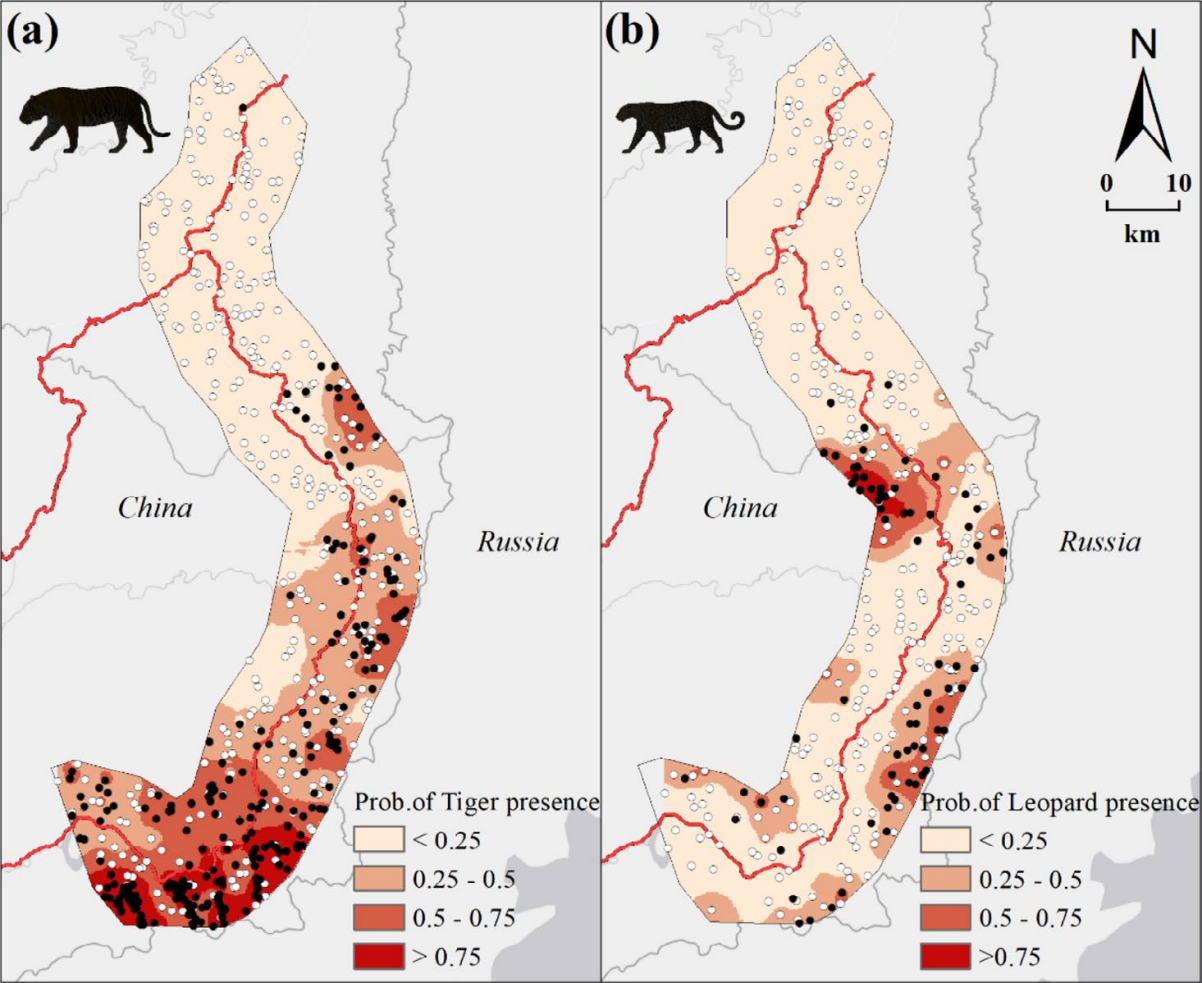
The presence of tigers (a) and leopards (b) in the study area was predicted using logistic geographically weighted regression (GWR) models. Filled circles represent their presence samples from field data, while unfilled circles indicate pseudo‐absence sample.

The logistic GWR accurately predicted the distribution patterns of Amur tigers and Amur leopards in our study area (Figure 7), showing that there is a higher occurrence probability of Amur tigers in the southern region, with only a few other areas on the eastern side of roads in the central region with high occurrence probabilities. However, this probability remains significantly low throughout the northern region. Amur leopards also avoid the northern region, and, even in the central and southern regions, the occurrence probability of Amur leopards increases only in areas where the occurrence of Amur tigers was relatively low.

## 4 Discussion

In this study, a combination of GLM model and logistic GWR model was employed to analyze species' spatial heterogeneity of habitat selection. Consistent with previous applications of GWR (Windle et al. 2010; Ye et al. 2020), our GWR results explicitly demonstrate the importance of spatial heterogeneity and non‐stationary directional changes in species‐environment relationships for Amur tigers, Amur leopards, roe deer, and wild boar. Additionally, visualizing local coefficient surfaces through GIS based on the GWR models highlights the relative distribution patterns of predators and prey across space concerning multiple variables.

### 4.1 Repercussions of Roads on Habitat Selection by Predators and Prey

A strong correlation was observed between the spatial distribution patterns of Amur tigers, Amur leopards, roe deer, and wild boar, and their proximity to roads of varying levels. This relationship underscores the potential impact that road networks can exert on these animal populations. The presence of roads can significantly influence wildlife movement and behavior (Forman and Alexander 1998). Proximity to primary highways notably influences their distribution across the study area; however, the effects of secondary and tertiary roads vary among regions. These findings suggest that highways with high volumes of traffic play a pivotal role in shaping predator–prey dynamics (Quiles and Barrientos 2024).

Interestingly, roe deer and wild boar prefer areas near highways during winter, which aligns with previous studies suggesting that certain prey species may choose to remain close to human habitats to avoid encounters with predators that tend to avoid humans (Carter et al. 2012). This behavior is particularly evident in caribou ( *Rangifer tarandus* ) populations inhabiting regions with higher predator densities, such as grizzly bears ( *Ursus arctos horribilis* ) (Berger 2007). Meanwhile, species such as roe deer are more likely to prefer areas adjacent to highways during winter due to the clearance of snow, which facilitates easier access to food resources and enhances their mobility (Torres et al. 2011). In contrast, large predators such as tigers and leopards exhibit a clear aversion of human activities; they actively evade primary roads (Carter et al. 2012). This discovery is not a surprise since previous studies have shown species‐specific responses of carnivores and ungulates to road density (DeCesare et al. 2012; Frair et al. 2008). These findings further highlight how high‐density human interference can indirectly benefit large herbivore species that are less sensitive to humans by reducing the presence of carnivores nearby (Muhly et al. 2011).

In addition to the disparity in tertiary road usage during winter, roe deer and wild boars exhibit diametrically opposite behaviors in their preferences. Wild boar demonstrate a significant inclination toward these roads across the entire region as they utilize them for movement and foraging (Johann et al. 2020). Due to their substantial size and great physical strength, wild boar can easily and expeditiously traverse these roads, gaining access to abundant food resources (D'Amico et al. 2016). Conversely, roe deer conspicuously avoid tertiary roads within the central area. While the availability of foraging opportunities may hold greater significance for wild boar compared to threats, this discrepancy does not apply to roedeer (Davidson et al. 2022; Torres et al. 2011).

### 4.2 Revealing Spatial Heterogeneity in Habitat Selection Within Predator–Prey Systems

Our logistic GWR model results align with the GLM analysis and previous non‐spatial models of resource selection of Amur tigers and leopards (Carroll and Miquelle 2006; Mcquillan and Rice 2015), while the local coefficient surface map reveals spatially varying effects of environmental variables. Notably, the correlations between environmental factors, such as mining areas and primary roads, and the distribution of Amur tigers are spatially consistent, detected by GLMs. The above‐mentioned correlations from the GWR models are spatially inconsistent. The logistic GWR model indicated that they had positive and negative relationships over the study area, a pattern not reported by previous Amur tiger research. Although actual disturbance by mining occupies a small land area (< 1% of the world's surface), influences of mining can occur at the landscape level and broad geographic scale (Bridge 2004). The variation of cross‐space coefficients primarily suggests that the distribution of Amur tigers is influenced by changing environmental factors, which can be attributed to the spatial heterogeneity of these variables, emphasizing the importance of factoring spatial heterogeneity when studying interactions between Amur tigers and their environment (Zhang et al. 2022).

Spatial distribution of the Amur leopard was also driven by the spatial heterogeneity of influencing environmental factors. The main determinants in the northern region were aspect, and distance to settlements and tertiary roads. Conversely, in the southern region, distance to primary roads and farmlands play a crucial role in determining their distribution. Furthermore, R_S_Road was found to impact habitat selection of leopards in the northern region, but not in the southern region. This may suggest that other factors also affect habitat selection patterns of Amur leopard within the southern regions (Farhadinia et al. 2021).

### 4.3 Distribution of the Amur Tiger and Leopard Across Linear Infrastructure Under Spatial Non‐Stationarity

Numerous SDMs have been established for tigers and leopards (Carroll and Miquelle 2006; Jiang et al. 2014; Kafley et al. 2019; Qi et al. 2015), but none of these models considered spatial heterogeneity or interactions between environmental variables. Through its advanced spatial analysis techniques, the GWR model considers the inherent spatial heterogeneity within different regions (Fotheringham et al. 2009). Incorporating spatial information into modeling can enhance identifying key driving factors contributing to spatial inconsistency in species‐environment relationships, thereby significantly improving the predictability of models in complex systems (Saefuddin et al. 2012). This means that it can capture subtle variations in habitat suitability for Amur tigers and leopards at local scale. As a result, the accurate distribution maps generated by the GWR model have significant implications for conservation efforts as they provide crucial information for wildlife managers and policymakers to identify priority areas for protection or restoration initiatives. In particular, conservation strategies can be tailored accordingly by pinpointing key locations where Amur tigers and leopards are more likely to occur or face threats from human activities (Meng et al. 2016). Furthermore, these maps serve as valuable tools for researchers studying population dynamics and ecological interactions involving Amur tigers and leopards (Carroll and Miquelle 2006). For example, the precise visualization of their distribution patterns allows scientists to investigate factors influencing animal movements, assess potential conflicts with humans living nearby, or even explore possible corridors connecting fragmented populations (Xiaofeng et al. 2011).

Spatial distribution patterns of the Amur tigers and leopards also indicated the barrier effect of the primary road on their spatial movement. Even in the southern region, where the occurrence probability of Amur tigers is generally high, the occurrence probability on the west side of the road is significantly lower than that on the east side. Most of the high occurrence probability areas of the Amur leopard is located only on one side of the road. However, the spatial heterogeneity of species‐environment relationships also reduces the sensitivity of these two big cats to roads in specific areas, for instance, tigers in the southern region and leopards in the central region. This provides key information for alleviating the obstruction posed by roads through conservation projects such as ecological corridor construction. The information presented here offers significant value for the conservation planning of the Amur tigers and leopards, especially in addressing their challenge of traversing Route 331 from Russia into China.

While GWR performs well in spatial relationship analysis, it should be used with caution as there are some shortcomings because geographical coordinates are the only information required to estimate local coefficients at unobserved locations (Fotheringham et al. 2003). First, due to local regression coefficients estimated based on the neighborhood observations, GWR cannot be used to predict species distribution outside the study area. Second, Li et al. (2018) mentioned that this method is unsuitable for predicting species' future distribution under substantial changes in environmental conditions. Third, the possible collinearity in local regression coefficients may limit the interpretation of species‐environment relationships (Wheeler and Tiefelsdorf 2005). Fourth, the prediction accuracy of GWR is sensitive to data quantity (Chen et al. 2012). Additionally, we acknowledge that the study would undoubtedly be refined by including additional explanatory variables affecting predator–prey distribution such as human density and traffic volume. Nevertheless, our straightforward analysis demonstrates the promising potential of GWR as a valuable tool for incorporating spatial heterogeneity into ecological analyses to understand spatial non‐stationarity's underlying causes and consequences in ecological processes.

## 5 Conclusions

By Integrating the logistic GWR model together with GLM model to assess the spatial distribution of Amur tigers, leopards, and their ungulate preys, our study highlights the prevalence of spatial heterogeneity in species‐environment relationships. This spatial heterogeneity may be manifested in the changes of key driving factors and the specific effects. In this context, our study has uncovered the local heterogeneity effects of habitat use by ungulate prey, which serve as critical predictors of habitat selection and distribution patterns of large felids. This provides new perspective to facilitate the development of tailored conservation strategies based on localized heterogeneity effects toward managing harmonious coexistence between local communities and wildlife in diverse areas.

## Author Contributions

**Xuankai Liang:** conceptualization (lead), data curation (lead), investigation (lead), methodology (lead), software (lead), writing – original draft (lead), writing – review and editing (equal). **Zexu Long:** conceptualization (lead), data curation (equal), formal analysis (equal), methodology (equal), writing – original draft (equal), writing – review and editing (equal). **Shiyu Chen:** data curation (equal), formal analysis (supporting), methodology (supporting), writing – review and editing (supporting). **Jinzhe Qi:** conceptualization (supporting), data curation (equal), writing – review and editing (equal). **Buyi Sun:** writing – original draft (equal), writing – review and editing (supporting). **Nathan James Roberts:** data curation (supporting), writing – review and editing (supporting). **Heng Bao:** writing – review and editing (supporting). **Guangshun Jiang:** conceptualization (lead), funding acquisition (equal), writing – review and editing (equal).

## Conflicts of Interest

The authors declare no conflicts of interest.

## Data Availability

All code and data are publicly available at Dryad. https://doi.org/10.5061/dryad.47d7wm3p6

## Appendix A

**TABLE A1:** Description of environmental variables employed in the modeling of predator and prey distribution.

Habitat factor	Habitat variable	Description of the habitat factor	Source	Units
Topographic factor	Elevation	Elevation grid with 200 m resolution	STRM product, (https://doi.org/10.5066/F7PR7TFT)	m
	Aspect	Aspect grid with 200 m resolution derived from the digital elevation model above	ditto	°
	Slope	Slope grid with 200 m resolution derived from the digital elevation model above	ditto	°
Forest type	BDF	Distance from the center point to the nearest closed (> 40%) broadleaved deciduous forest patch	GlobCover 2009 product of the European Space Agency (http://due.esrin.esa.int/page_globcover.php)	m
	NDEF	Distance from the center point to the nearest open (15%–40%) needle‐leaved deciduous or evergreen forest patch	ditto	m
	MBNF	Distance from the center point to the nearest closed to open (> 15%) mixed broadleaved and needle‐leaved forest patch	ditto	m
	MFSG	Distance from the center point to the nearest mosaic forest or shrubland (50%–70%) / grassland (20%–50%) patch	ditto	m
	MGFS	Distance from the center point to the nearest mosaic grassland (50%–70%)/forest or shrubland (20%–50%) patch	ditto	m
	SV	Distance from the center point to the nearest Sparse (< 15%) vegetation patch	ditto	m
Human disturbance	Settlements	Distance from the center point to the nearest temporary settlements	National Basic Geographic Database, version 2015, 1:250000 (https://www.ngcc.cn/ngcc/)	m
	Farmland	Distance from the center point to the nearest farmland	ditto	m
	Ginseng	Distance from the center point to the nearest ginseng land	ditto	m
	Mining	Distance from the center point to the nearest mining area	ditto	m
	Pasture	Distance from the center point to the nearest pasture	ditto	m
	Village	Distance from the center point to the nearest village	ditto	m
	P _ Road	Distance from the center point to the nearest primary road	ditto	m
	S _ Road	Distance from the center point to the nearest secondary road	ditto	m
	T _ Road	Distance from the center point to the nearest tertiary road	ditto	m
Weather factor	Snow	Average snow depth within a 200 m grid	Transect line survey in January and February 2018	cm
Other	Food	Number of branches of the current year's shrubs in a 200 m grid	ditto	—
	River	Distance from the center point to the nearest river. The river includes the primary river and its tributaries	National Basic Geographic Database, version 2015, 1:250000 (https://www.ngcc.cn/ngcc/)	m

**TABLE A2:** The coefficients for explanatory variables in both the global logistic regression model (GLM) and logistic Geographically Weighted Regression (logistic GWR) models were estimated to analyze predator and prey distribution patterns.

Species	Predictors	GLM					Logistic GWR				
		Coefficient estimate	Confidence interval			*p*	Minimum	Lower quartile	Median	Upper quartile	Maximum
			2.50%	97.50%							
Roe deer	(Intercept)	−0.53	−0.71	−0.36		< 0.001	−1.128	−0.726	−0.619	−0.567	−0.439
	P_Road	−0.70	−0.90	−0.51	−	< 0.001	−0.947	−0.923	−0.858	−0.504	−0.433
	S_Road	0.21	0.03	0.38	+	0.020	0.080	0.109	0.134	0.258	0.360
	T_Road	0.17	−0.01	0.36	*	0.067	0.080	0.127	0.152	0.178	0.366
	Food	0.24	0.05	0.43	+	0.012	0.104	0.162	0.273	0.286	0.368
	BDF	−0.17	−0.36	0.01	*	0.064	−0.375	−0.306	−0.128	−0.106	−0.068
	MBNF	−0.27	−0.46	−0.09	−	0.003	−1.228	−0.542	−0.285	−0.269	−0.123
	MFSG	0.27	0.10	0.45	+	0.003	0.242	0.258	0.288	0.321	0.370
Wild boar	(Intercept)	−0.51	−0.94	−0.10		0.017	−0.764	−0.710	−0.615	−0.553	−0.531
	Settlements	−0.44	−0.95	0.03	*	0.078	−0.650	−0.579	−0.519	−0.494	−0.471
	Pasture	−0.78	−1.45	−0.21	−	0.013	−1.259	−1.174	−0.914	−0.674	−0.464
	P_Road	−0.92	−1.43	−0.48	−	< 0.001	−1.207	−1.157	−1.130	−0.959	−0.901
	T_Road	−0.84	−1.40	−0.35	−	0.001	−1.072	−0.986	−0.781	−0.735	−0.674
	Snow	0.83	0.42	1.28	+	< 0.001	0.798	0.836	0.844	0.874	0.920
	NDEF	0.42	−0.05	0.92	*	0.085	0.249	0.351	0.380	0.482	0.601
	MGFS	−0.58	−1.13	−0.08	−	0.028	−0.863	−0.775	−0.593	−0.494	−0.481
Tiger	(Intercept)	−0.75	−1.03	−0.50		< 0.001	−2.590	−1.752	−1.248	−0.165	2.237
	Slope	−0.38	−0.59	−0.18	−	< 0.001	−0.491	−0.391	−0.354	−0.309	−0.204
	Farmland	−0.53	−0.82	−0.25	−	< 0.001	−1.019	−0.882	−0.801	−0.474	−0.160
	Mining	0.89	0.48	1.33	+	< 0.001	−6.759	0.007	1.205	2.561	4.510
	P_Road	0.31	0.09	0.55	+	0.007	−0.586	−0.094	0.218	0.587	0.684
	NDEF	0.22	0.00	0.45	*	0.055	−0.609	−0.322	0.436	0.549	0.785
	R_MBNF	0.47	0.12	0.82	+	0.009	−0.703	0.285	1.192	2.297	9.246
	W_Snow	−1.28	−1.90	−0.72	−	< 0.001	−10.399	−2.709	−1.880	−1.226	0.507
Leopard	(Intercept)	−2.93	−3.91	−2.16		< 0.001	−3.327	−3.292	−3.091	−2.823	−2.681
	Aspect	0.44	0.12	0.79	+	0.009	0.396	0.437	0.514	0.561	0.572
	Settlement	−2.13	−3.29	−1.07	−	< 0.001	−2.596	−2.553	−2.329	−2.038	−1.872
	Farmland	0.77	0.41	1.16	+	< 0.001	0.607	0.613	0.704	0.772	0.803
	P_Road	0.65	0.30	1.02	+	< 0.001	0.596	0.623	0.671	0.674	0.677
	T_Road	−0.51	−0.92	−0.14	−	0.010	−0.906	−0.872	−0.650	−0.510	−0.454
	R_S_Road	−0.96	−2.09	−0.05	−	0.063	−1.446	−1.326	−1.161	−0.841	−0.673

*Note:* Slope indicates slope, Aspect indicates aspect, P _ Road indicates distance to primary road, S _ Road indicates distance secondary road, T _ Road indicates distance to tertiary road, Food indicates food abundance: number of branches of the current year's shrubs in a 200 m grid. Pasture indicates the distance to pasture, Settlements indicate the distance to temporary settlements, and Snow indicates snow depth: Average snow depth within a 200 m grid. Farmland indicates the distance to farmland, BDF indicates the distance to a closed broadleaved deciduous forest patch, MBNF indicates the distance to closed to open mixed broadleaved and needle‐leaved forest patch, MFSG indicates the distance to mosaic forest or shrubland/grassland patch, NDEF indicates the distance to open needle‐leaved deciduous or evergreen forest patch, MGFS indicates the distance to close to mosaic grassland/forest or shrubland patch, R_MBNF indicates the local coefficient of the MBNF variable in the logistic GWR model for roe dee distribution, W _ Snow indicates the local coefficient of the Snow variable in the logistic GWR model for wild boar distribution, R _ S _ Road indicates the local coefficient of the S _ Road variable in the logistic GWR model for roe dee distribution. A negative correlation is denoted by "−", a positive correlation is indicated by "+", and random influence is represented by "*".
